# dcHiChIP: a comprehensive Nextflow-based pipeline for multiscale analysis of chromatin architecture from HiChIP data

**DOI:** 10.1093/bioinformatics/btag636

**Published:** 2026-09-12

**Authors:** Abhishek Agarwal, Ziad Al Bkhetan, Dariusz Plewczynski

**Affiliations:** University of Warsaw, Centre of New Technologies, Laboratory of Functional and Structural Genomics, Banacha 2c, Warsaw, Mazovia, 02-097, Poland; Australian BioCommons, The University of Melbourne, North Melbourne, VIC 3051, Australia; University of Warsaw, Centre of New Technologies, Laboratory of Functional and Structural Genomics, Banacha 2c, Warsaw, Mazovia, 02-097, Poland; Warsaw University of Technology, Faculty of Mathematics and Information Science, Laboratory of Bioinformatics and Computational Genomics, Warsaw, Mazovia, 00-662, Poland

## Abstract

**Motivation:**

Despite the growing use of HiChIP to investigate protein-directed chromatin architecture, a comprehensive and reproducible pipeline for analysing these datasets-from raw reads to multiscale 3D genome features-remains lacking. Existing tools often focus on isolated components, such as loop calling or matrix generation, but fall short in integrating structural annotation, functional enrichment, and spatial modeling within a unified framework. To address this gap, we developed dcHiChIP, a modular, scalable Nextflow-based workflow that streamlines the analysis of HiChIP data, enabling both routine processing and in-depth exploration of chromatin organization and regulatory interactions.

**Results:**

dcHiChIP enables robust and reproducible analysis of HiChIP datasets across multiple scales of chromatin architecture. It accepts raw sequencing data as input and generates high-quality loop calls, domain annotations, and 3D genome models. It also performs functional annotation and motif enrichment analyses. Applied to benchmark CTCF HiChIP datasets, dcHiChIP identifies major chromatin architectural features such as TADs/CCDs, A/B compartments, and chromatin stripes, and offers efficient, end-to-end execution with support for batch processing and workflow resumability.

**Availability:**

dcHiChIP is publicly available on GitHub at https://github.com/SFGLab/dcHiChIP, with documentation at https://sfglab.github.io/dcHiChIP/. The software version used in this study is archived at Zenodo: https://doi.org/10.5281/zenodo.22030542

## 1 Introduction

The spatial organization of the genome within the nucleus plays a crucial role in regulating gene expression, developmental processes, and cellular identity (Bonev and Cavalli 2016, Dekker and Mirny 2016, Furlong and Levine 2018, Rowley and Corces 2018). Chromatin looping, topologically associating domains (TADs), A/B compartments, and other three-dimensional (3D) structures facilitate interactions between regulatory elements, such as enhancers and promoters, over long genomic distances. To investigate these complex genomic interactions, high-throughput chromosome conformation capture (3C)-based techniques have emerged as indispensable tools. Among them, HiChIP combines the power of chromatin immunoprecipitation (ChIP) and Hi-C to selectively enrich for chromatin contacts mediated by specific proteins and chromatin marks such as CTCF, Cohesin, and histone modifications, thereby increasing resolution and efficiency while reducing sequencing costs (Mumbach *et al.* 2016). Despite the growing adoption of HiChIP and related technologies, their comprehensive analysis remains technically demanding. Current workflows rely on numerous uncoordinated scripts and independently developed tools that must be adapted to diverse input formats, genome builds, and experimental contexts. Moreover, many existing pipelines lack modularity and reproducibility and do not integrate structural and functional genomic annotations across multiple scales. These limitations pose significant barriers to users without extensive computational experience, constraining the reproducibility and scalability of chromatin architecture research.

Several recent efforts have attempted to address the challenges of HiChIP data analysis. Tools such as HiC-Pro (Servant *et al.* 2015), MAPS (Juric *et al.* 2019), and FitHiChIP (Bhattacharyya *et al.* 2019) have introduced workflows for detecting chromatin loops. However, most of these tools focus on a narrow analytical scope—typically limited to loop calling—and lack support for downstream integrative features such as chromatin domain annotation, 3D reconstruction, or transcription factor motif analysis. Moreover, many of these tools are not designed for reproducible, scalable execution across high-performance computing (HPC) or cloud environments.

As part of these ongoing efforts, our group previously developed nf-HiChIP, a targeted workflow optimized for Cohesin HiChIP data, which enabled robust detection of chromatin loops and linear stripes (Buka *et al.* 2025). While this protocol significantly improved interaction signal quality and provided a streamlined analysis path for Cohesin-associated datasets, it was tailored to specific experimental settings and lacked a fully modular structure or the capability to support multiscale architectural feature extraction across diverse HiChIP profiles.

While several tools and workflows have been developed for individual components of HiChIP analysis, they typically focus on specific tasks such as read processing, contact matrix generation, or loop detection. The novelty of dcHiChIP lies not in developing a new loop-calling algorithm, but in integrating established HiChIP and 3D genome analysis tools into a unified, modular, reproducible, and scalable Nextflow DSL2 workflow (Table S1). By combining data processing, loop detection, multiscale analysis of chromatin architecture, functional annotation, reproducibility assessment, and 3D genome modeling within a single framework, dcHiChIP enables comprehensive end-to-end analysis from raw sequencing data to biological interpretation.

dcHiChIP is a comprehensive and flexible pipeline that integrates pre-processing, contact map generation, peak and loop detection, and multiscale architectural analysis—including the identification of TADs/CCDs, A/B compartments, sub-compartments, and linear chromatin stripes. It further supports functional annotation, motif enrichment, aggregate peak analysis (APA), and sample correlation assessment, enabling end-to-end exploration of the 3D chromatin landscape. The pipeline also includes a dedicated module for 3D genome modeling, enabling users to reconstruct chromatin conformations at gene, chromosome, or genome scales. With support for batch processing, portable computing environments, and robust documentation, dcHiChIP provides a scalable, reproducible solution for comprehensive HiChIP data analysis across diverse biological contexts.

## 2 Methods

### 2.1 Pipeline overview

**Figure 1:**
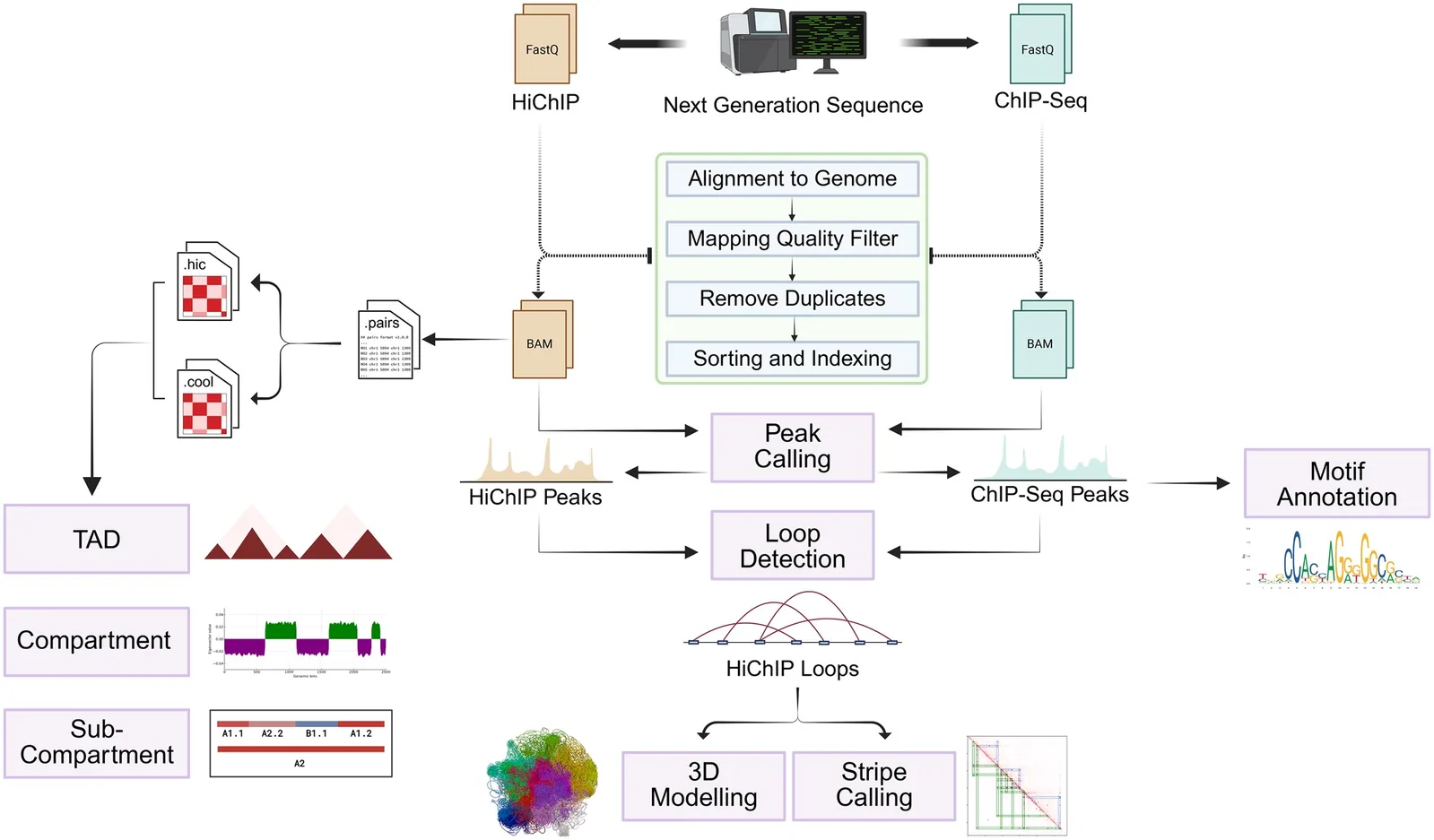
Overview of the dcHiChIP workflow for multiscale chromatin architecture analysis. The pipeline integrates HiChIP and matched ChIP-seq inputs, performing alignment, quality filtering, duplicate removal, and BAM indexing before peak calling and loop detection. Downstream analyses include TAD/CCD and compartment identification, sub-compartment annotation, stripe calling, 3D genome modeling, and transcription factor motif enrichment. The workflow produces a unified, multi-resolution view of chromatin organization from raw FASTQ files to final 3D architectural features.

dcHiChIP is a reproducible, scalable analysis pipeline developed with Nextflow (Di Tommaso *et al.* 2017) for end-to-end analysis of HiChIP and ChIP-seq data. A simplified diagram of the pipeline is included in Fig. 1. It generates high-resolution chromatin contact maps, loop calls, structural feature identification, and functional annotation from raw FASTQ files. The pipeline follows nf-core community best practices and can be easily customized and executed on various computing environments. The pipeline takes paired-end HiChIP data and, optionally, ChIP-seq reads and peak files in BED format as input. If narrow peaks are provided as input, they will be used throughout the analysis, and the peak-calling step will be skipped. If no peaks are provided as input, ChIP-seq data will be used for peak calling. Otherwise, the whole analysis will be carried out on HiChIP data. This happens automatically in the workflow based on the available data in the input sample sheet. The codebase and documentation are freely available at https://github.com/SFGLab/dcHiChIP and https://sfglab.github.io/dcHiChIP/, respectively.

By default, dcHiChIP generates multi-resolution contact matrices at 1 kb, 2 kb, 5 kb, 10 kb, 20 kb, 50 kb, 100 kb, 200 kb, and 500 kb resolutions. A/B compartment and sub-compartment analyses are performed at 100 kb resolution by default. The workflow is distributed with hg38 reference resources but can be readily adapted to other genome assemblies by providing the corresponding reference genome, annotation, chromosome size, blacklist, and genome feature files. Detailed descriptions of all configurable parameters and default settings are available in the online documentation.

The tools integrated into dcHiChIP were selected based on their widespread adoption within the 3D genomics community, demonstrated performance on HiChIP or related chromatin conformation datasets, active maintenance, and compatibility with reproducible workflow execution in containerized environments. Importantly, dcHiChIP was implemented using a modular Nextflow DSL2 architecture, allowing individual components to be replaced, extended, or supplemented as new methods emerge. This design enables a user to incorporate alternative tools for loop detection, chromatin architecture analysis, and 3D genome modeling without major modifications to the overall workflow. The main analytical stages implemented in dcHiChIP are described below.

### 2.2 Read alignment

Read alignment is performed using BWA-MEM (Li and Durbin 2009).

### 2.3 Peak calling

To identify chromatin-binding sites that serve as anchors for interaction analysis, dcHiChIP performs peak calling using either HiChIP-derived reads or, if available, paired ChIP-seq data. When ChIP-seq is unavailable, the pipeline uses HiChIP reads directly and applies MACS3 (Zhang *et al.* 2008) to identify significant peaks representing protein-bound chromatin regions. If ChIP-seq reads are available, they are aligned and processed in parallel, and MACS3 is again used to define narrowPeak regions. Alternatively, users may supply precomputed narrowPeak files for downstream loop-calling.

### 2.4 Loop detection

Chromatin loops are detected using a modified implementation of the MAPS algorithm (Juric *et al.* 2019). The modifications are limited to workflow-level performance optimization and input handling. Computationally intensive preprocessing steps, including read alignment, sorting, duplicate removal, and mate information correction using samtools fixmate, were moved out of MAPS and parallelized within the dcHiChIP Nextflow workflow. The resulting binary alignment/map format (BAM) file is supplied to MAPS in its expected input format. The input-handling logic was also modified to support chromosome-restricted analyses when data for some chromosomes are absent. No changes were made to the MAPS loop-detection algorithm, statistical model, significance-testing procedure, or false discovery rate (FDR) control.

### 2.5 Generation of multi-resolution contact matrices

Read pairs are parsed using Pairtools (Open2C, Abdennur *et al*. 2024b), and multi-resolution contact matrices are generated in Juicer-compatible .hic and Cooler (Abdennur and Mirny 2020) compatible .mcool format.

### 2.6 Identification of topologically associating domains, chromatin contact domains, A/B compartments, sub-compartments and hierarchical domain inference, and chromatin stripes

dcHiChIP detects TADs using an insulation score-based method implemented in Cooltools (Open2C, Abdennur *et al.* 2024a). Chromatin Contact Domains calling is performed using our in-house scripts, which have been previously tested for HiChIP and ChIA-PET datasets. A/B compartments are calculated by applying principal component analysis (PCA) to Pearson correlation matrices derived from distance-normalized interaction frequencies obtained from cooler contact matrices. dcHiChIP utilizes CALDER2 (Liu *et al.* 2021) for sub-compartment inference, which detects multi-level domain structures through recursive segmentation of the genome based on local interaction patterns and hierarchical clustering. By default, sub-compartment analysis is performed at 100 kb resolution using the recommended CALDER2 settings, although the user can modify these parameters in the pipeline configuration file. Chromatin stripes are then identified using gStripe (Buka *et al.* 2025) from cooler contact matrices.

### 2.7 Functional annotation and motif enrichment

Loop anchors and peak regions are annotated using HOMER (Heinz *et al.* 2010) and Bioframe (Abdennur *et al.* 2024c), and then intersected with genomic features such as promoters, exons, introns, and intergenic elements defined in Gene Transfer Format (GTF) files. Motif enrichment analysis is performed on anchor regions using the HOMER FindMotifsGenome tool and motif libraries from JASPAR (Castro-Mondragon *et al.* 2022). This allows the identification of overrepresented transcription factor binding motifs associated with chromatin loops and regulatory domains.

### 2.8 Signal quantification, quality control, and reproducibility metrics

Genome-wide coverage of aligned reads is calculated using deepTools bamCoverage (Ramírez *et al.* 2016), generating BigWig files for visualization and downstream quantification. Signal reproducibility across samples is assessed using multiBigWigSummary, followed by plotCorrelation and plotPCA modules from deepTools. Sample-specific correlation matrices, principal component plots, and loop overlap statistics provide quantitative metrics of biological variation and replicate consistency. Coverage plots are also generated using a custom deepTools wrapper for genome-wide inspection of signal distribution.

### 2.9 Three-dimensional (3D) genome modeling

dcHiChIP includes an integrated module for chromatin structure reconstruction using the MultiMM framework (Korsak *et al*. 2024), which enables 3D modeling of genome organization based on experimentally derived chromatin loops and A/B compartment annotations. Spatial restraints are constructed from loops identified during dcHiChIP analysis and translated into distance restraints between interacting genomic loci. Compartment annotations provide additional constraints reflecting large-scale genome organization. MultiMM then uses these restraints to generate ensembles of three-dimensional conformations consistent with the observed interaction patterns. Users can generate models at different resolutions, ranging from individual gene loci to whole chromosomes or entire genomes, depending on the density of input loops and matrix coverage. Output models are exported in .cif or compatible formats and can be visualized in tools such as UCSF Chimera or PyMOL.

## 3 Results

### 3.1 Implementation, flexibility, and reproducibility of dcHiChIP

dcHiChIP is implemented in Nextflow DSL2, enabling the smooth, parallel, and automated execution of multiple tasks across local machines, HPC clusters, and cloud computing environments. The workflow supports containerization technologies, including Docker, Singularity, and Apptainer, as well as Conda environments. It is configured with all its required dependencies and data resources and requires minimal user input. To demonstrate its flexibility and applicability to different experimental designs, dcHiChIP was evaluated across three common usage scenarios: (i) processing only HiChIP FASTQ files, (ii) processing HiChIP FASTQ files together with user-provided ChIP-seq peak files (narrowPeak format), and (iii) joint processing of raw HiChIP and ChIP-seq FASTQ files for integrated peak calling and chromatin interaction analysis (Supplementary Figs. S1–S3).

### 3.2 Performance evaluation

The dcHiChIP workflow benefits from the parallel execution framework provided by Nextflow, which enables independent modules and sample-level processes to run concurrently on high-performance computing systems. To evaluate the computational performance and applicability of the pipeline, we processed publicly available GM12878 CTCF HiChIP datasets, SRR7990656 and SRR7990657, together with matched ChIP-seq datasets, SRR998175 and SRR998176. In addition, to assess the applicability of dcHiChIP beyond CTCF-associated chromatin loops, we processed the GM12878 Cohesin HiChIP datasets SRR3467175, SRR3467176, SRR3467177, and SRR3467178 for loop calling and reproducibility analysis (Fig. S4).

To further support dcHiChIP’s ability to recover expected chromatin interaction features, we performed loop overlap benchmarking against established loop-calling methods and published reference loop datasets. Specifically, dcHiChIP-derived CTCF and Cohesin loops were compared with loops identified by FitHiChIP, HiCCUPS, and HG00731 reference loop datasets. The benchmarking results are summarized in Fig. S5, which reports the total number of loops used for each comparison and shows pairwise loop overlap percentages using a paired-anchor overlap strategy. These analyses showed concordance between dcHiChIP loop calls and established or published reference loop sets, supporting the consistency of dcHiChIP loop calls with these reference sets.

**Figure 2:**
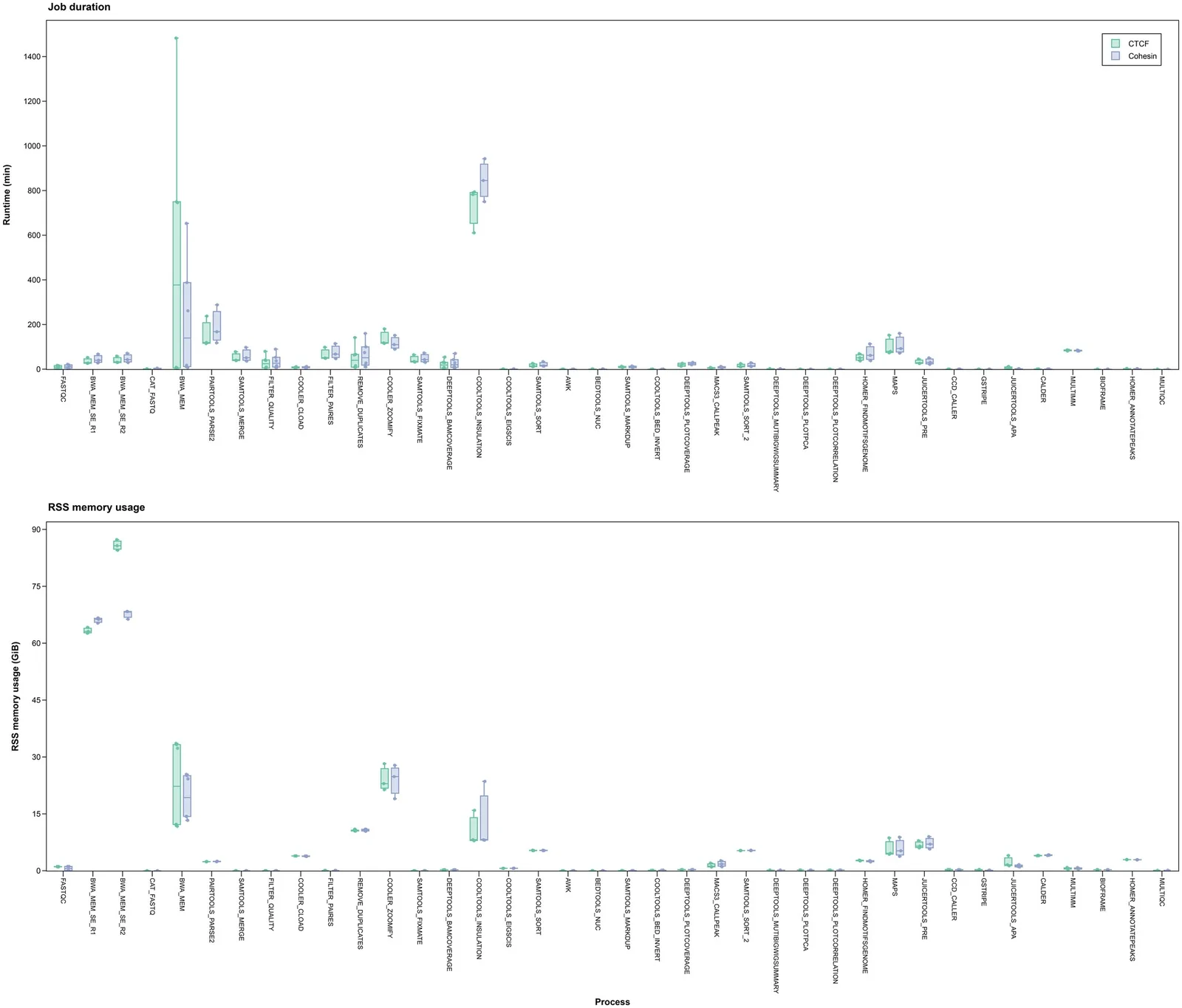
Runtime and memory usage of the dcHiChIP workflow modules during processing of the GM12878 CTCF and Cohesin HiChIP datasets. The upper panel shows job duration for each workflow module, reported in minutes, whereas the lower panel shows peak resident set size (RSS), reported in GiB. CTCF and Cohesin task executions are shown in green and blue, respectively. Boxplots summarize the distribution of measurements across individual task executions for each module; the center line indicates the median, the box represents the interquartile range, and the whiskers extend to values within 1.5 times the interquartile range. Individual task measurements are overlaid as points. Peak RSS is the maximum amount of physical memory used during the execution of an individual task.

Benchmark analyses were performed on an HPC cluster equipped with Intel Xeon Platinum 8274 Cascade Lake processors operating at 3.2 GHz. Processes were executed as independent jobs using Singularity containers under Linux and the PBS Professional scheduler. The maximum default resource request for an individual process was 12 CPU cores and 72 GB of RAM; for retried tasks, requests could increase to a maximum of 16 CPU cores and 128 GB of RAM. Figure 2 summarizes the runtime and peak memory requirements for each module of the dcHiChIP workflow. The end-to-end elapsed time for processing the GM12878 CTCF and Cohesin HiChIP datasets was approximately 44 hours and 34 hours, respectively, on the HPC system, including scheduler queue time. As shown, the most time- and memory-intensive stages are BWA-MEM alignment, Cooltools insulation, and cooler zoomify, which together dominate the workflow’s computational load. Other steps, such as read pairing, fragment assignment, and loop annotation, execute efficiently with low memory usage. MultiMM is also computationally demanding; however, in this benchmark, it was run on a single chromosome to illustrate its resource profile. These results characterize the computational requirements of dcHiChIP on an HPC system and identify its most resource-intensive modules, helping users plan cluster resource allocation.

To illustrate the 3D genome modeling capabilities of dcHiChIP, chromatin interaction data from the GM12878 CTCF HiChIP dataset were used to generate representative models at gene-level, regional, chromosome, and genome-wide organizational scales using MultiMM (Fig. S6). The resulting models illustrate how experimentally derived chromatin interactions and compartment annotations can be integrated to reconstruct three-dimensional genome organization.

## 4 Discussion

dcHiChIP provides an optimized, end-to-end workflow for HiChIP data, integrating alignment, peak calling, loop detection, and structural annotation within a unified Nextflow framework. In contrast to workflows that rely on multiple standalone tools and ad-hoc scripts, dcHiChIP offers a modular design that adapts to diverse experimental setups, protein targets, and computational environments, supporting both routine analyses and advanced chromatin-architecture studies.

A central strength of dcHiChIP lies in its comprehensive analytical scope. The pipeline implements the MAPS algorithm for statistical loop detection (Juric *et al.* 2019) and produces multi-resolution contact matrices in .hic and .mcool formats compatible with visualization and analysis tools such as Juicebox and Cooltools (Durand *et al.* 2016, Open2C, Abdennur *et al*. 2024b). Integrated modules perform loop and anchor annotation, motif enrichment (HOMER and JASPAR), and multiscale structural characterization, including TAD identification, A/B compartment and sub-compartment classification (CALDER2), and stripe detection (gStripe). Together, these components enable the systematic interrogation of both local and global chromatin organization across various conditions or cell types.

Beyond structural analysis, dcHiChIP extends to 3D genome modeling through integration with MultiMM (Korsak *et al*. 2024). By combining loop and compartment data, the pipeline reconstructs chromatin conformations from gene-level to chromosome-scale resolutions, providing users with spatial insights into the regulatory architecture. Built with reproducibility and scalability at its core, dcHiChIP leverages Nextflow’s process tracking, containerization, and checkpoint-based resumability to ensure consistent performance from personal workstations to HPC clusters. Thus, dcHiChIP connects HiChIP read processing with structural analysis, 3D genome modeling, and functional annotation within a reproducible workflow.

## Supplementary Material

btag636_Supplementary_Data

## Data Availability

The sequencing datasets used in this study are publicly available in the NCBI Sequence Read Archive (SRA) under accession numbers SRR7990656, SRR7990657, SRR998175, SRR998176, SRR3467175, SRR3467176, SRR3467177, and SRR3467178. The dcHiChIP source code is publicly available on GitHub at https://github.com/SFGLab/dcHiChIP, with documentation at https://sfglab.github.io/dcHiChIP/. The software version used in this study is archived at Zenodo at https://doi.org/10.5281/zenodo.22030542.
