# Supplementary material for: dcHiChIP: a comprehensive Nextflow-based pipeline for multiscale analysis of chromatin architecture from HiChIP data
Source: Bioinformatics. 2026 Sep 12;42(9):btag636. doi: 10.1093/bioinformatics/btag636 (PMC13576066; doi:10.1093/bioinformatics/btag636)
Supplement: Supplementary file 1 [file btag636_supplementary_data.pdf]

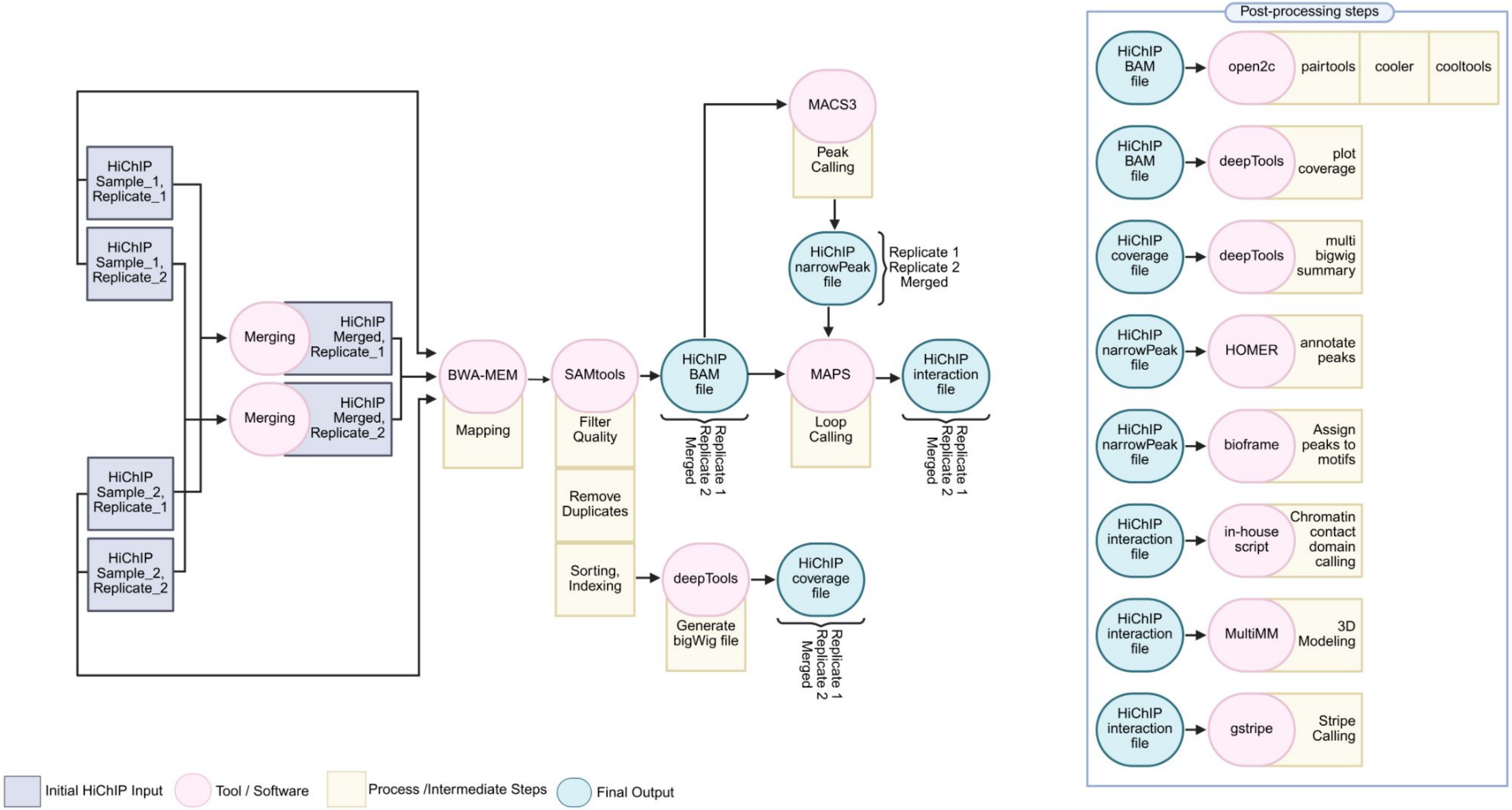

Supplementary Figure S1. Workflow of dcHiChIP for analysis of HiChIP FASTQ files only. ChIP-enriched regions are identified directly from the processed HiChIP data using MACS3 before loop calling with MAPS. Downstream analyses include coverage generation, motif annotation, chromatin contact domain calling, stripe detection, and 3D genome modeling.

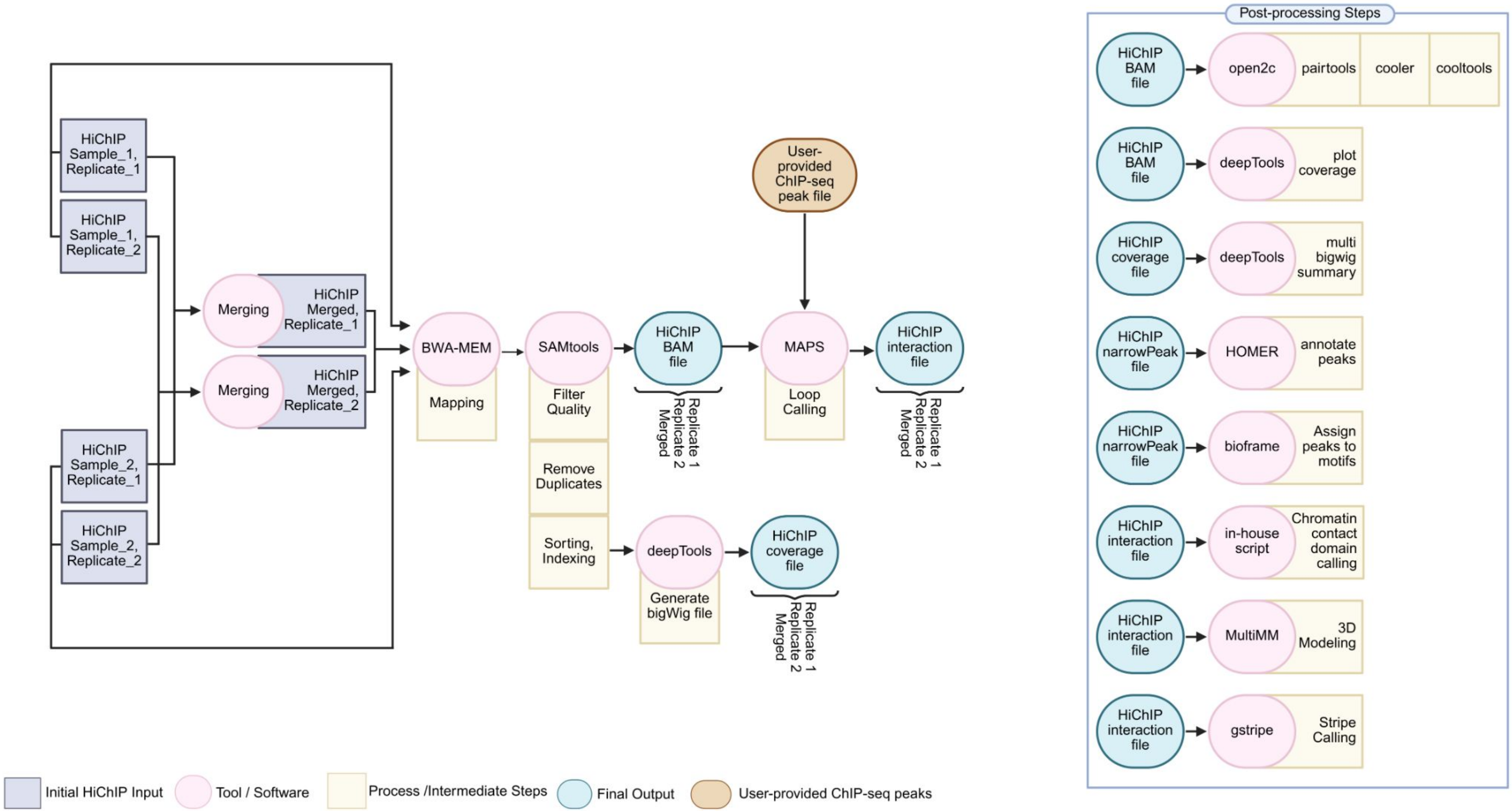

Supplementary Figure S2. Workflow of dcHiChIP using raw HiChIP FASTQ files together with user-provided ChIP-seq peak files (narrowPeak format). User-supplied peak calls are incorporated directly into MAPS for loop detection, bypassing the ChIP-seq processing and peak-calling steps.

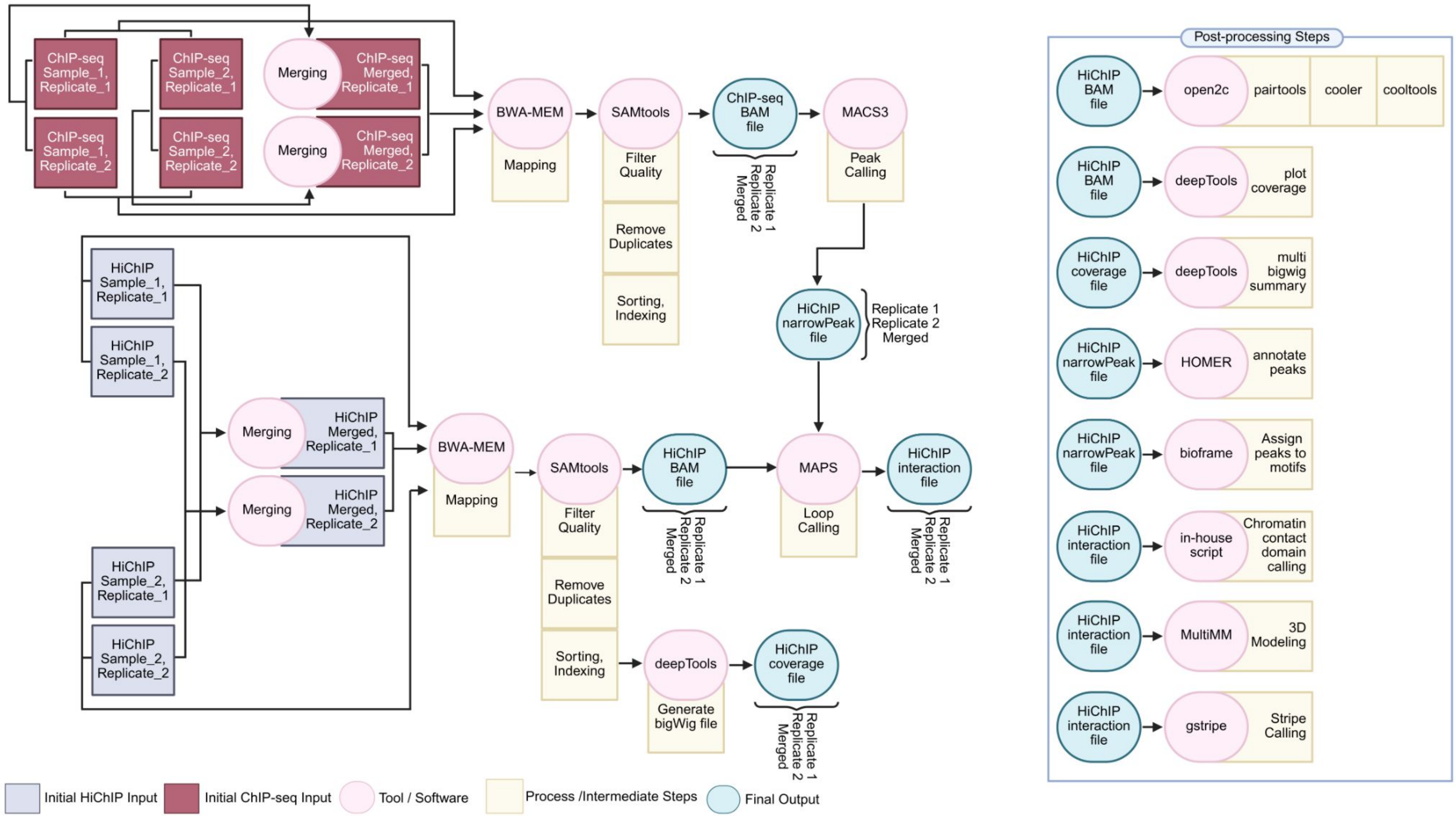

Supplementary Figure S3. Workflow of dcHiChIP for the integrated analysis of raw HiChIP and ChIP-seq FASTQ files. The workflow performs read processing, alignment, quality filtering, ChIP-seq peak calling using MACS3, loop detection using MAPS, and downstream analyses including coverage generation, motif annotation, chromatin contact domain calling, stripe detection, and 3D genome modeling.

(A)

Sample	Accession Number(s)	Loops
CTCF Replicate 1	SRR7990656	74397
CTCF Replicate 2	SRR7990657	57984
Cohesin Replicate 1	SRR3467175	7291
	SRR3467176	
Cohesin Replicate 2	SRR3467177	8421
	SRR3467178	

(B)

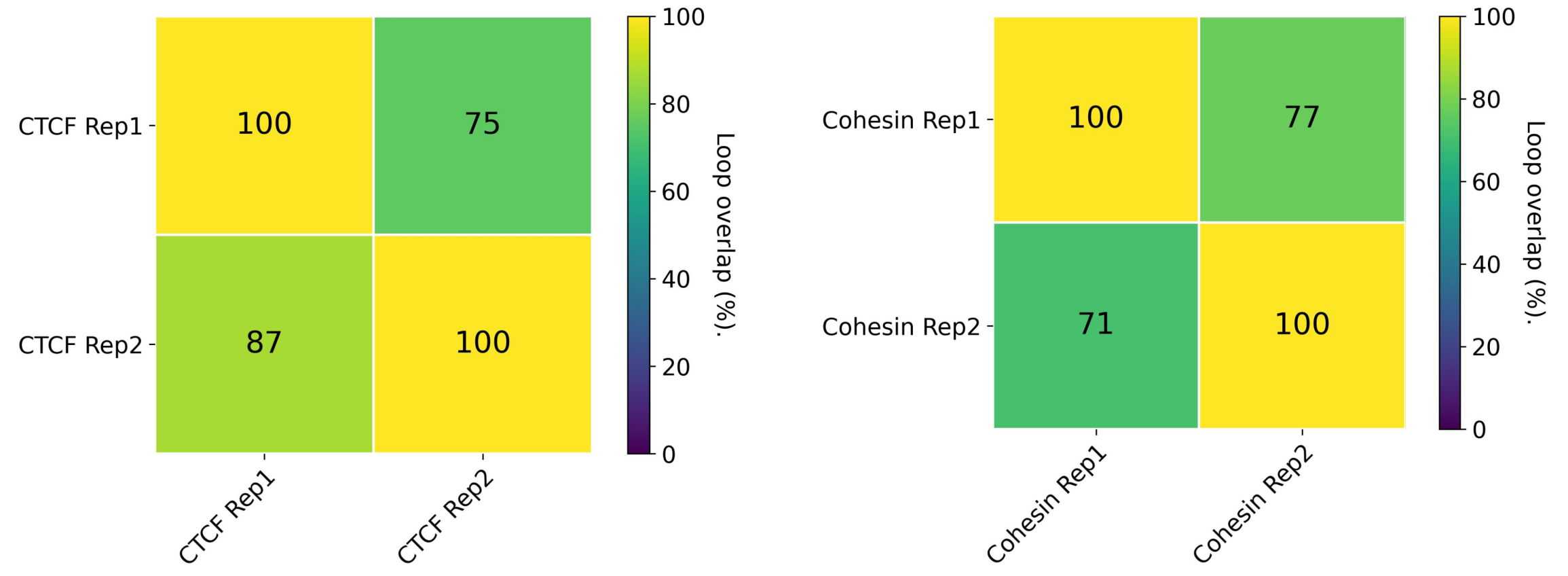

(C)

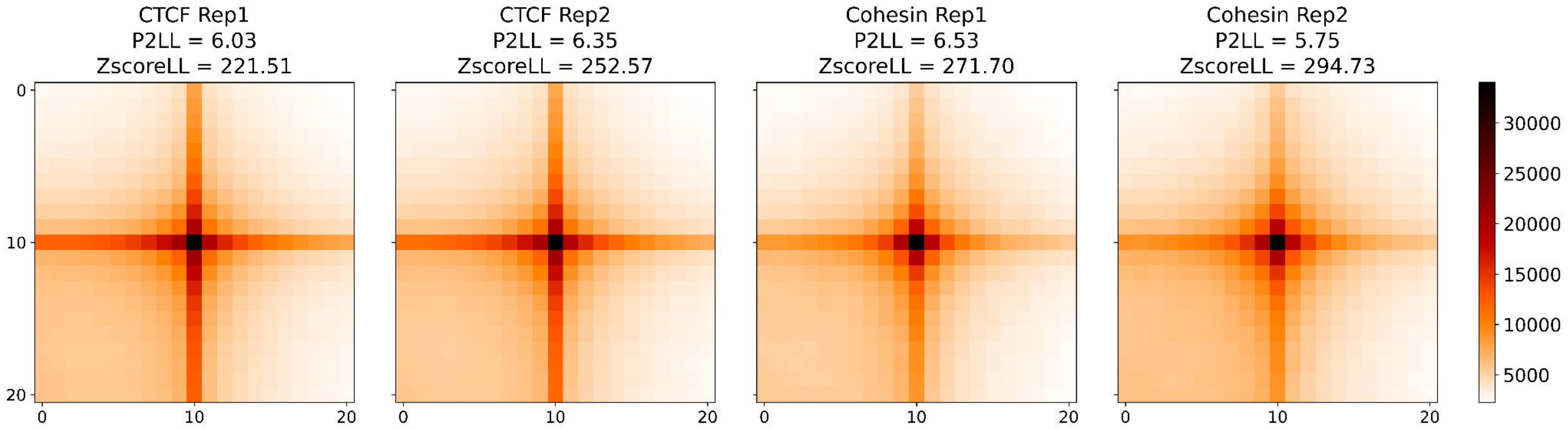

Supplementary Figure S4. Reproducibility and aggregate enrichment of dcHiChIP loop calls in CTCF and Cohesin HiChIP datasets. (A) HiChIP datasets for CTCF and Cohesin used for loop calling by the dcHiChIP pipeline. (B) Pairwise overlap between loops called by dcHiChIP in the indicated datasets. Each cell in the matrix represents the percentage of loops in the row sample that overlap one or more loops in the column sample. The matrix is not symmetric because each percentage is calculated using the total number of loops in the row sample as the denominator, and one-to-many or many-to-one overlaps may occur. (C) Aggregate peak analysis (APA) of loops identified in each replicate. APA matrices were generated genome-wide at 5-kb resolution for each sample. APA enrichment was quantified using the peak-to-lower-left background ratio (P2LL) and lower-left background z-score (ZscoreLL), both calculated from the central pixel relative to the lower-left background region.

(A)

Category*	Loop source	CTCF	Cohesin
**Pipeline**	dcHiChIP	179325	36662
	FitHiChIP	202613	49045
	HiCCUPS	59754	19478
**Sample**	HG00731	28012	80487

*Pipeline refers to loop sets generated using loop-calling tools, whereas Sample refers to published reference loop datasets used for comparison.

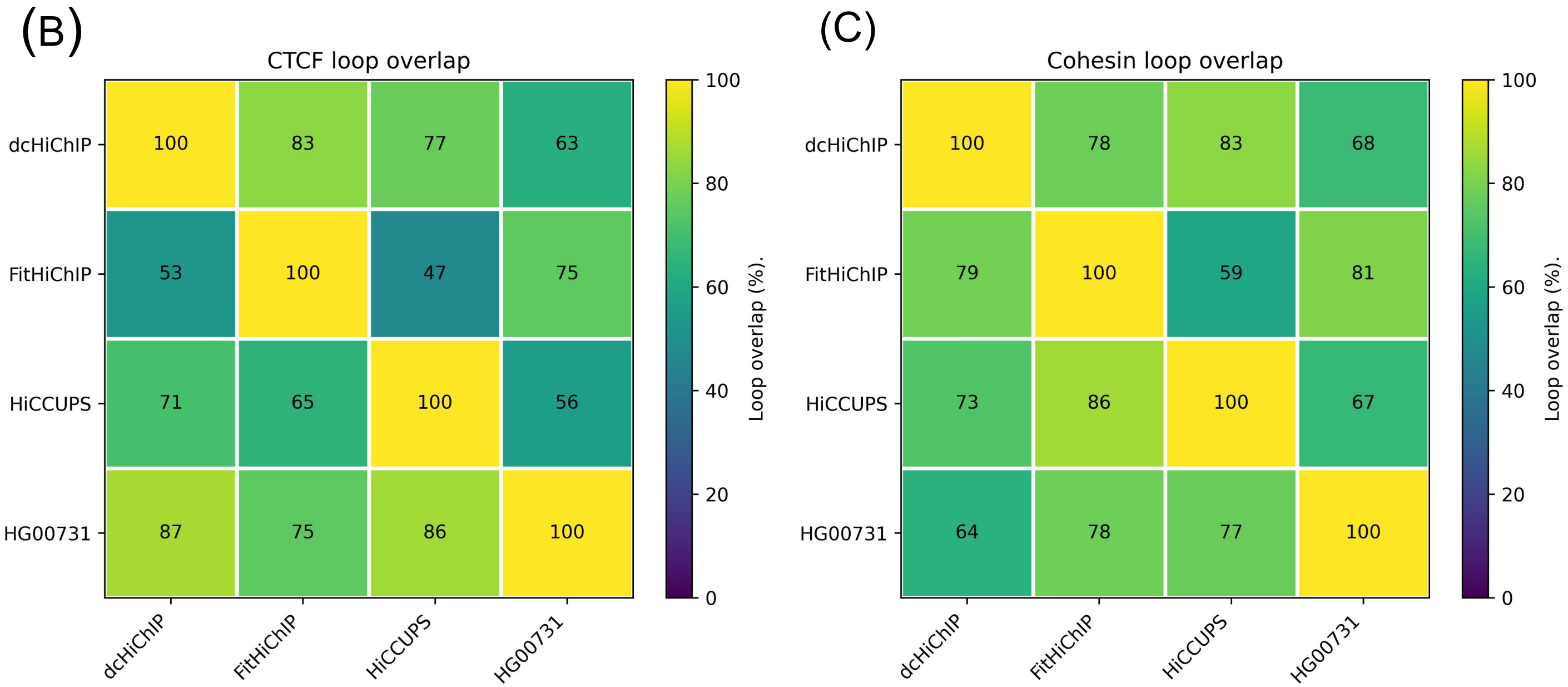

Supplementary Figure S5. (A) The table reports the number of CTCF and Cohesin/SMC1 loops used for benchmarking across dcHiChIP, FitHiChIP, HiCCUPS, and HG00731 reference datasets. Heatmaps show pairwise loop overlap percentages for (B) CTCF and (C) Cohesin loops. Each cell represents the percentage of loops in the row dataset that overlap one or more loops in the column dataset using a paired-anchor overlap strategy. The matrices are not symmetric because each percentage is calculated using the total number of loops in the row dataset as the denominator, and one-to-many or many-to-one overlaps may occur. dcHiChIP loops overlapped with loops identified using established loop-calling methods and with published reference loop datasets.

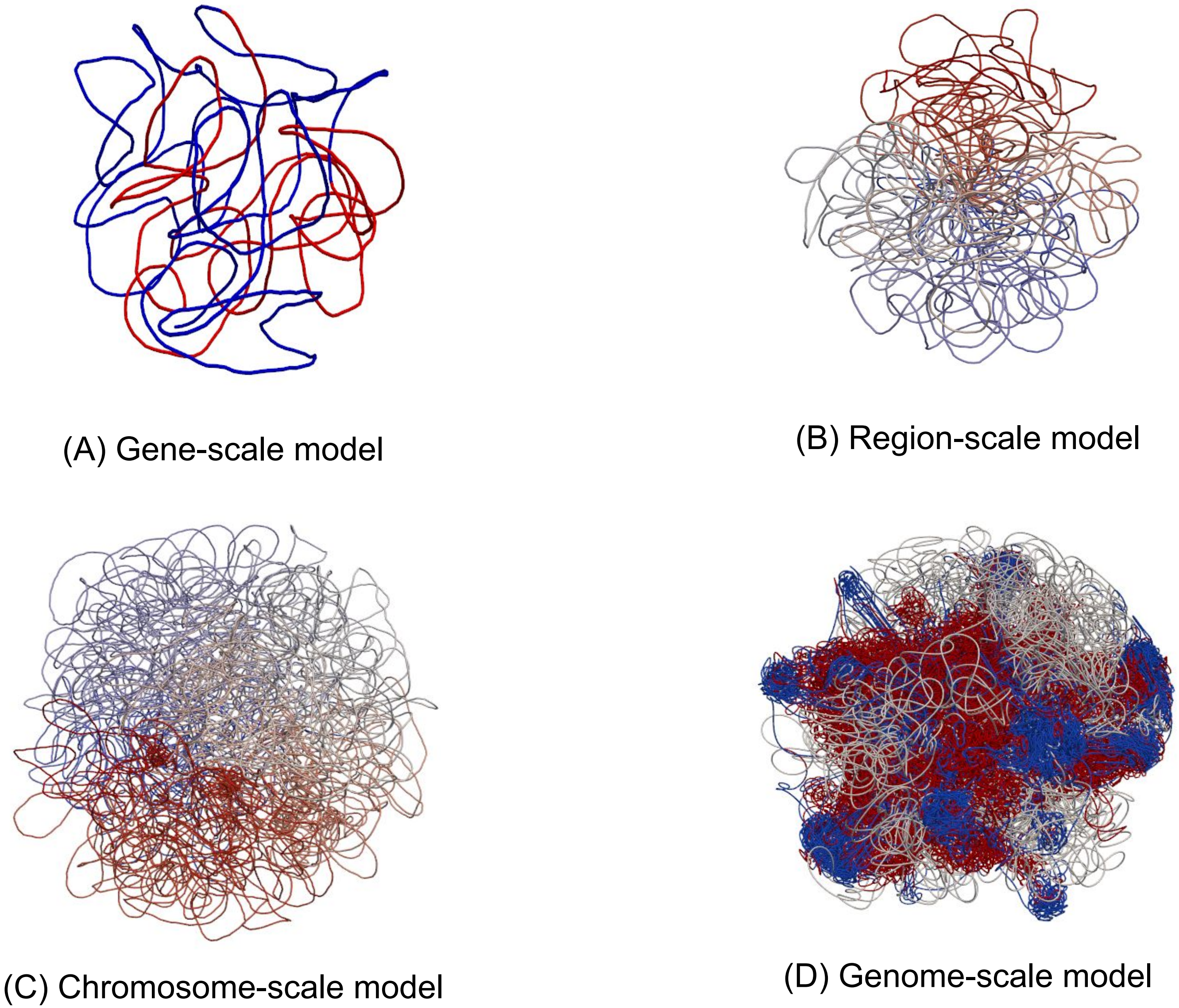

Supplementary Figure S6. Representative MultiMM-based 3D genome models generated through dcHiChIP using the GM12878 CTCF HiChIP dataset. (A) Gene-scale model centered on the PIRAT1 locus. (B) Region-scale model of chr8:10,949,488-11,360,500. (C) Chromosome-scale model of chromosome 21. (D) Genome-wide chromatin model reconstructed from HiChIP-derived loop interactions. Spatial restraints were derived from chromatin loops identified by dcHiChIP and translated into distance restraints between interacting genomic loci. These restraints were used by MultiMM to generate three-dimensional genome conformations consistent with the observed interaction patterns.

Feature	dcHiChIP	MAPS	FitHiChIP	nf-core/hic	HiC-Pro	nf-HiChIP
Nextflow workflow	✓	✗	✗	✓	✗	✓
DSL2 modular architecture	✓	✗	✗	✓	✗	✗
Container support (Docker/Singularity/Apptainer)	✓	✗	✗	✓	✗	✓
HiChIP-specific analysis	✓	✓	✓	✗	✗	✓
Processing from raw FASTQ files	✓	✗	✗	✓	✓	✓
User-supplied peak support	✓	✓	✓	✗	✗	✓
Loop calling	✓	✓	✓	✗	✗	✓
Contact matrix generation (.hic/.mcool)	✓	✗	✗	✓	✓	✓
TAD/CCD detection	✓	✗	✗	✓	✓	✗
A/B compartment analysis	✓	✗	✗	✓	✓	✗
Sub-compartment analysis	✓	✗	✗	✗	✗	✗
Chromatin stripe detection	✓	✗	✗	✗	✗	✗
Motif enrichment analysis	✓	✗	✗	✗	✗	✗
Functional genomic annotation	✓	✗	✗	✗	✗	✗
Aggregate peak analysis (APA)	✓	✗	✗	✗	✗	✗
3D genome modeling	✓	✗	✗	✗	✗	✗
Workflow resumability	✓	✗	✗	✓	✗	✓
End-to-end analysis from raw data to biological interpretation	✓	✗	✗	Partial	Partial	Partial

*Partial indicates that the tool provides some relevant outputs but does not perform the complete analysis as an integrated workflow.

Supplementary Table S1. Comparison of dcHiChIP with widely used Hi-C and HiChIP analysis tools, including MAPS, FitHiChIP, HiC-Pro, nf-core/hic, and nf-HiChIP. The table summarizes supported features related to workflow modularity, chromatin interaction analysis, structural annotation, functional enrichment, and downstream 3D genome modeling.
